# *Oreocharis zijinensis* (Gesneriaceae), a new species from Guangdong, China

**DOI:** 10.3897/phytokeys.281.204573

**Published:** 2026-11-04

**Authors:** Guo-Li Zhu, Chi Xiong, Ye-Chun Xu, Jian-Jun Tan, Zi-Bing Xin, Xiao-Mao Qian, Ke Tan, Fang Wen, Xin-Xiang Bai

**Affiliations:** 1 College of Forestry, Guizhou University, CN-5500252, Guiyang, Guizhou, China College of Forestry, Guizhou University Guiyang China; 2 Guangxi Key Laboratory of Plant Conservation and Restoration Ecology in Karst Terrain, Guangxi Institute of Botany, Guangxi Zhuang Autonomous Region and Chinese Academy of Sciences, CN-541006, Guilin, Guangxi Zhuang Autonomous Region, China Guangxi Key Laboratory of Plant Conservation and Restoration Ecology in Karst Terrain, Guangxi Institute of Botany, Guangxi Zhuang Autonomous Region and Chinese Academy of Sciences Guilin China; 3 National Gesneriaceae Germplasm Resources Bank of GXIB, Gesneriad Committee of China Wild Plant Conservation Association, Gesneriad Conservation Center of China (GCCC), Guangxi Institute of Botany, Guilin Botanical Garden, Guangxi Zhuang Autonomous Region and Chinese Academy of Sciences, CN-541006, Guilin, Guangxi Zhuang Autonomous Region, China National Gesneriaceae Germplasm Resources Bank of GXIB, Gesneriad Committee of China Wild Plant Conservation Association, Gesneriad Conservation Center of China (GCCC), Guangxi Institute of Botany, Guilin Botanical Garden, Guangxi Zhuang Autonomous Region and Chinese Academy of Sciences Guilin China; 4 Hubei Provincial Key Laboratory for Protection and Application of Special Plant Germplasm in Wuling Area of China, Key Laboratory of State Ethnic Affairs Commission for Biological Technology, College of Life Sciences, South-Central Minzu University, CN-430074, Wuhan, Hubei, China Hubei Provincial Key Laboratory for Protection and Application of Special Plant Germplasm in Wuling Area of China, Key Laboratory of State Ethnic Affairs Commission for Biological Technology, College of Life Sciences, South-Central Minzu University Wuhan China; 5 Environmental Horticulture Research Institute/Guangdong Provincial Key Laboratory of Ornamental Plant Germplasm Innovation and Utilization, Guangdong Academy of Agricultural Sciences, CN-510640, Guangzhou, Guangdong, China Environmental Horticulture Research Institute/Guangdong Provincial Key Laboratory of Ornamental Plant Germplasm Innovation and Utilization, Guangdong Academy of Agricultural Sciences Guangzhou China

**Keywords:** Gesneriaceae, morphology, new taxon, *Oreocharis acaulis*, phylogeny

## Abstract

*Oreocharis zijinensis*, a new species discovered in Guangdong, China, is described and illustrated. The new species is morphologically similar to *O. acaulis* in the corolla shape and color, and the staminodes number, but is easily distinguished by leaf blade shape and indumentum, lateral veins number, the shape of corolla tube and lobes, and the length of stamens and staminodes. Phylogenetic analysis illustrates that the new species is nested within *Oreocharis*. Both morphological and molecular evidence support the identification of *O. zijinensis* as a new species. At present, only one population was confirmed in Dongjiang Forest Farm, Zijin County, with about 800 mature individuals. According to the IUCN Red List Categories and Criteria, it is provisionally assessed as Vulnerable [VU].

## Introduction

The genus, *Oreocharis* Bentham (1876: 1021), was redefined and enlarged some years ago, and it has become a significant and morphologically diverse genus (Möller et al. 2011b, 2014; Chen et al. 2014). *Oreocharis* s.l. comprises approximately 155 species (excluding infraspecific taxa) distributed in the mountainous areas of southern China, with the Hengduan Mountains and its adjacent areas, the Wuling Mountains and the Nanling Mountains as its three diversity distribution centers, and a small number of species are distributed southward to the northern edge of the Indochina Peninsula and northward to Japan (Kong et al. 2022; GRC 2026), e. g. *O. konkakinhensis* T.V.Do & T.T.D.Pham (Pham et al. 2026), *O. sanglocii* K.S.Nguyen, Aver., Nuraliev, L.Wu & C.W.Lin (Nguyen et al. 2025). In the past three years, 8 new species of *Oreocharis* have been discovered and documented in China (Hu et al. 2023; Huang et al. 2023; Li et al. 2023, 2025; Xiong et al. 2023; Zhou et al. 2023; Xie et al. 2024; Liu et al. 2025).

**Figure 1.**
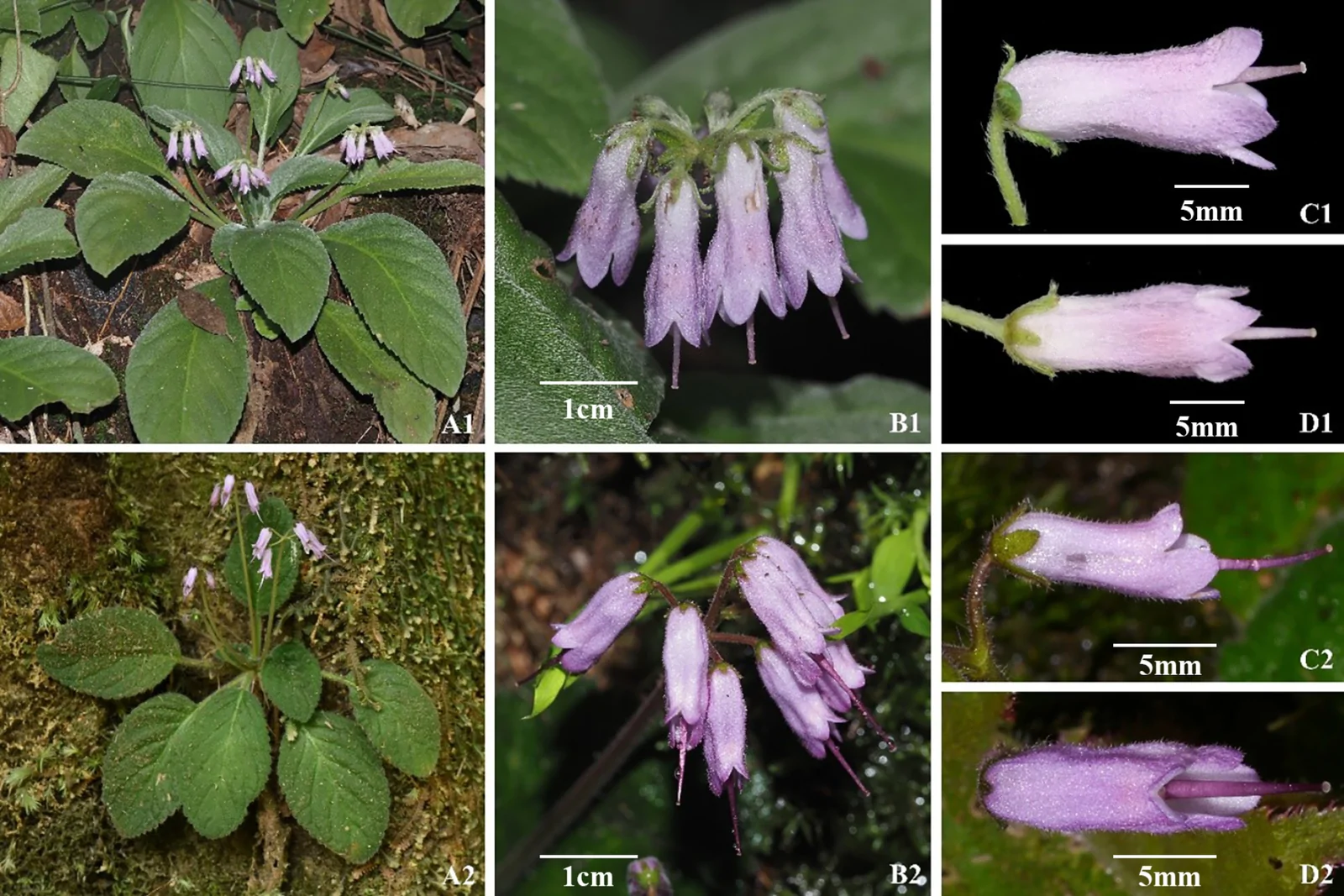
Morphological comparison of *Oreocharis zijinensis* sp. nov. (1) and *O. acaulis* (2). **A**. Habit; **B**. Inflorescence; **C**. Side view of a flower; **D**. Top view of a flower. **C1, D1** were photographed by Fang Wen, and others by Ye-Chun Xu.

In April 2021, during a plant survey in Zijin County, Guangdong, we encountered an unknown species of Gesneriaceae with leaves covered with silvery-white hairs. Some living plants were introduced and cultivated at the Gesneriad Conservation Center of China (GCCC) and the National Gesneriaceae Germplasm Bank for further study. In April 2024, we revisited the locality. We saw that this species was in the flowering stage with pink flowers, and confirmed that it is a member of *Oreocharis* according to the reproductive organ’s characters (viz., two fertile stamens, fused anthers; capsule straight in relation to pedicel, much longer than calyx, dehiscing loculicidally to base; valves 2, straight, not twisted). We carefully dissected the flowers and examined the relevant specimens and literature on the genus (Wang et al. 1990, 1998; Hu and Chen 2005; Wei et al. 2010; GRC 2026; POWO 2026). We confirmed that it represents a new species of *Oreocharis*, and its morphological characters are compared with the closely similar species *O. acaulis* (Fig. 1). The new species is described and illustrated below.

**Figure 2.**
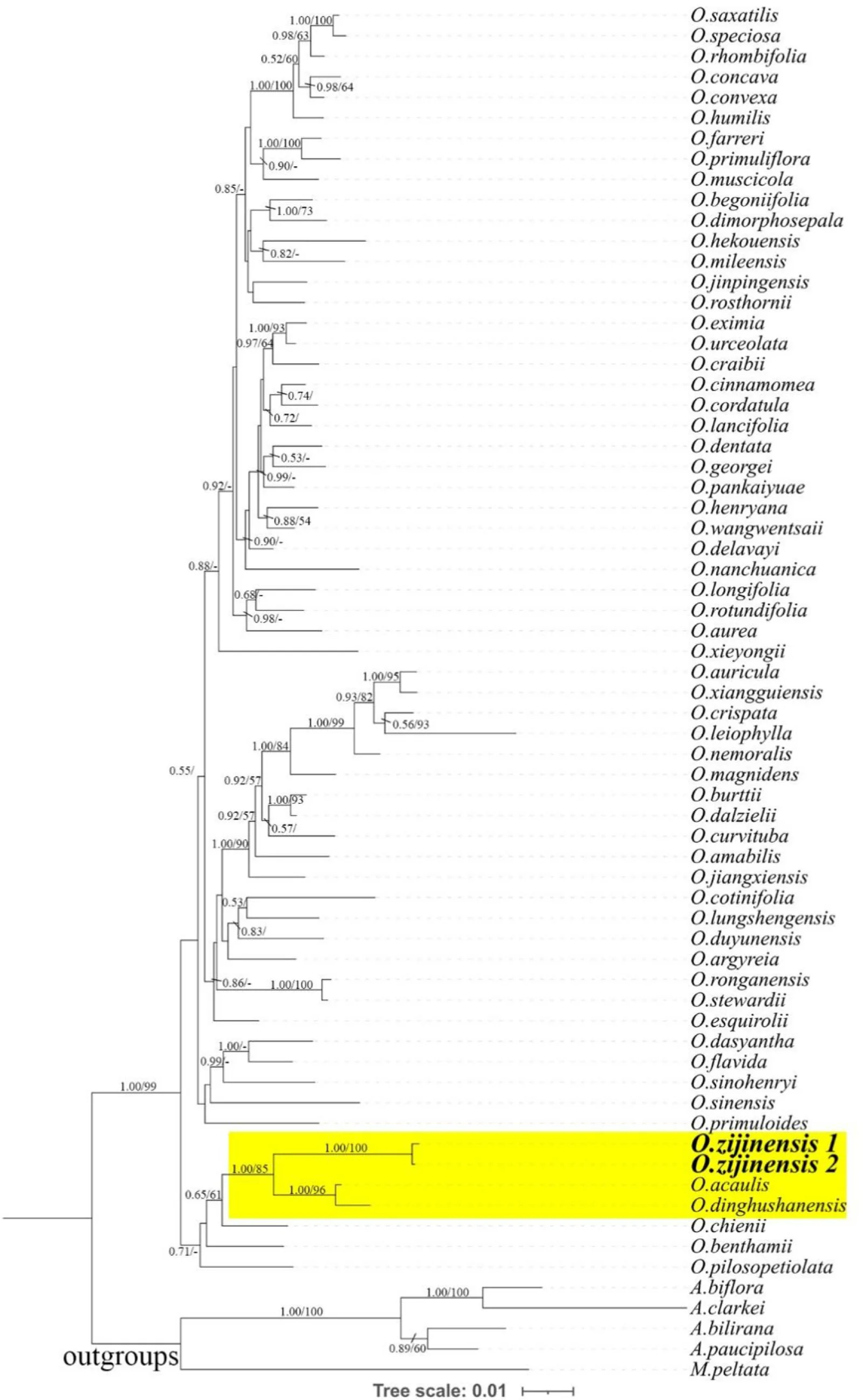
Bayesian 50% majority-rule consensus tree for *Oreocharis* inferred from combined *trn*L-F and ITS sequence data. Bayesian posterior probabilities are shown left branches and ML values are given right; “-” denotes branches with < 50% bootstrap support. The new species is shown in bold, with the branch on which it was placed marked in yellow.

The morphology of the new species was compared with that of similar taxa. Subsequently, phylogenetic reconstruction was performed using Maximum Likelihood and Bayesian Inference analyses to determine its systematic position. Collectively, morphological and molecular evidence supports the recognition of this taxon as a new species within *Oreocharis* (Table 1, Figs 1, 2).

**Table 1.** Comparison of morphological characters of *Oreocharis zijinensis* and *O. acaulis*.

**Characters**		***O. zijinensis***	***O. acaulis***
Leaf blades	Size	3–12 × 2–4 cm	1.7–7 × 1.1–3 cm
	Shape	elliptic to oblong-ovate, ovate	ovate to narrowly ovate
	Apex	Acute	acute to obtuse
	Base	attenuate to cuneate	broadly cuneate to rounded
	Symmetry	slightly asymmetric	nearly symmetric
	indumentum	adaxially densely silvery-white pubescent, abaxially white villous especially on the veins	adaxially densely brownish puberulent, abaxially sparsely puberulent
	lateral veins	5–10	4–5
Corolla	Color	pink at the apex and pale purple to white at the base	pink
	overall shape	cylindric in outline, not laterally compressed	oblong-cylindric in outline, strongly laterally compressed
	total length (including the corolla limb)	12–15 mm long	7–10 mm long
	tube shape	cylindric to narrowly campanulate, slightly enlarged at the mouth	strongly laterally compressed tubular, not swollen
	tube size	10–12 × 3–5 mm	6–8 × ca. 3 mm
Corolla limb	Shape	distinctly 2-lipped, apex acute	Indistinctly 2-lipped
	lobe shapes	equal, lobes triangular semiorbicular	ovate, apex orbicular
	lobes size	upper and lower lips nearly equal, adaxial lip 2.5–3 mm long; abaxial lip 1.5–2 mm long	lobes of upper and lower lips distinctly unequal, adaxial lip ca. 1 mm long; abaxial lip ca. 2.8 mm long
Stamens length		ca. 10 mm long	ca. 3.7 mm long
Staminodes		three, nearly equal, ca. 6.0 mm long	the adaxial one ca. 2.0 mm long, the abaxial ones ca. 3.2 mm long

## Materials and methods

### Morphological analysis

All specimens of the new species examined in this study were collected from Zijin County, Guangdong Province, China. Morphological characteristics were described based on both fresh plants and herbarium specimens. The new species was carefully compared with similar species in terms of morphology (Harris and Harris 2006; Beentje 2016). The terminology used to describe morphological characters followed the guidelines proposed by Harris and Harris (1994) and the Flora of China (Wang et al. 1990, 1998). Voucher specimens used for comparison are deposited in the Herbarium of Guangxi Institute of Botany, Chinese Academy of Sciences.

### DNA sequencing and molecular analysis

To reconstruct the phylogeny of *Oreocharis*, we sampled 61 species (62 samples with the new species represented by two accessions), encompassing the majority of those with available sequence data in GenBank. Following prior phylogenetic work (Möller et al. 2011a), *Agalmyla* Blume (four species) and *Metapetrocosmea peltata* (Merr. & Chun) W.T. Wang were selected as outgroups.

Species’ leaf samples collected from the place of origin were quickly dried with silica gel for DNA extraction (Chase and Hills 1991). Two primers and relevant PCR protocols were selected for DNA regions ITS and *trn*L*-*F following (Möller et al. 2011b). Sequences were aligned with MAFFT v.7.505 (Katoh and Standley 2013), and gap sites were removed with trimAl v1.2rev57 (Capella-Gutiérrez et al. 2009) using “-automated1” command. The dataset showed no significant substitution saturation (Iss < Iss.c, P = 0.0000) as assessed in DAMBE v5.3.19 (Xia 2013), confirming its suitability for phylogenetic analysis. The optimal partitioning scheme and substitution models were selected under the AICc using PartitionFinder2 (Lanfear et al. 2016). Maximum likelihood phylogenies were inferred using IQ-TREE v2.2.0 (Nguyen et al. 2015) under Edge-linked partition model for 1000 standard bootstraps. Bayesian Inference phylogenies were inferred using MrBayes v3.2.7a (Ronquist et al. 2012) under partition model. Phylogenetic trees were reconstructed using both maximum likelihood and Bayesian inference in PhyloSuite v.1.2.3 (Zhang et al. 2020; Xiang et al. 2023), with model testing conducted in PAUP* v4.0b10 (Swofford 2003).

Two independent MCMC analyses were conducted, each with four chains run for 10,000,000 generations and sampled every 1,000 generations. After discarding the first 25% of samples as burn-in, a 50% majority-rule consensus tree was generated from the combined post-burn-in trees. Stationarity was confirmed when the average standard deviation of split frequencies fell below 0.01. For maximum likelihood analyses, branch support was assessed with 1,000 bootstrap replicates. Trees and support values were visualized using iTOL v.6 (Letunic and Bork 2024).

## Results

### Morphological comparisons

**Figure 3.**
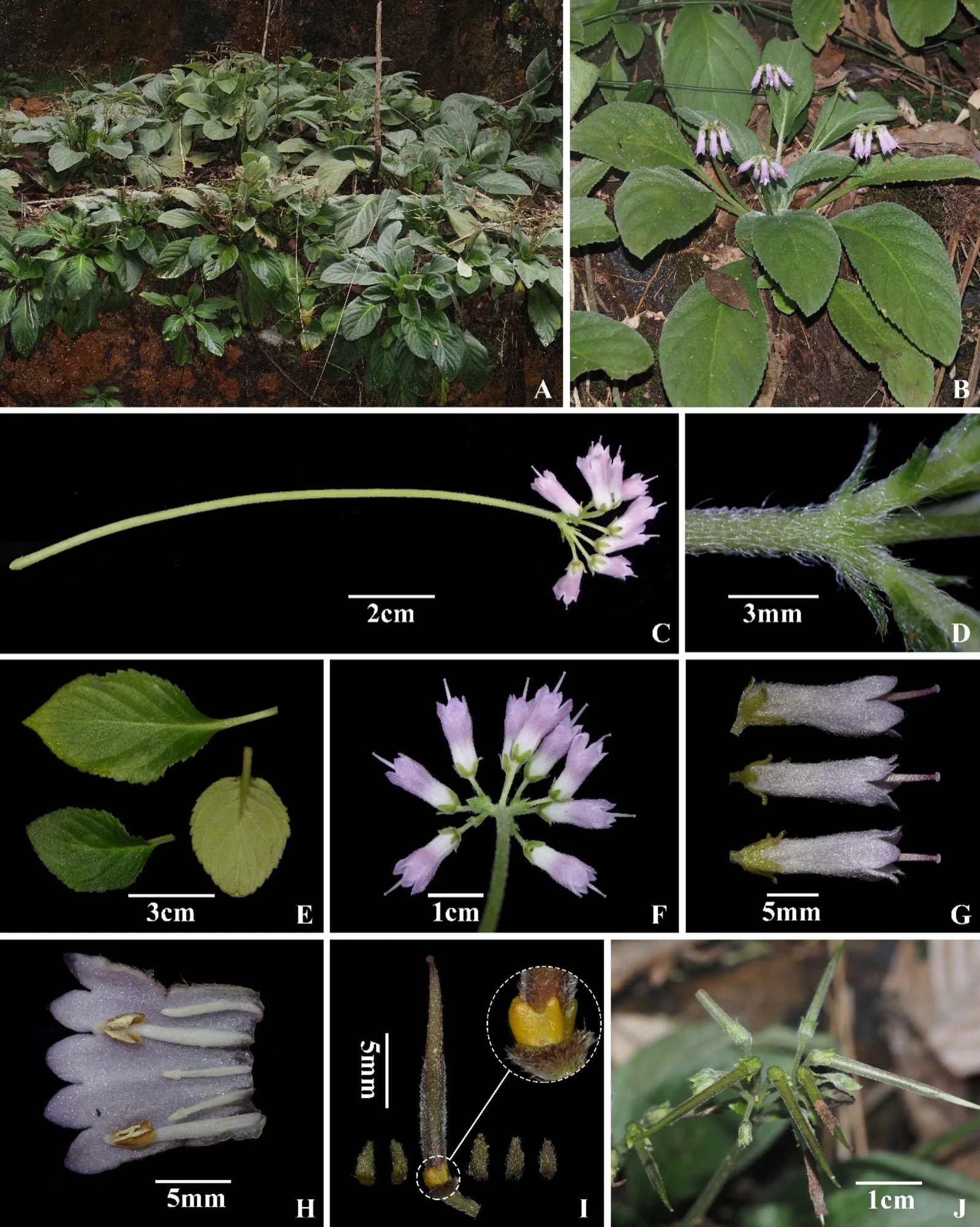
*Oreocharis zijinensis* sp. nov. **A**. Habitat; **B**. Habit; **C**. Inflorescence; **D**. Bracts; **E**. Leaf blades; **F**. Inflorescence; **G**. Flowers; **H**. Opened corolla showing stamens; **I**. Pistil with disc and calyx lobes; **J**. Capsules. **A, B, J** were photographed by Ye-Chun Xu, **C, D, F, H** by Fang Wen, and others by Chi Xiong.

The new species is morphologically similar to *Oreocharis acaulis* in the corolla shape and color, and the staminodes number. It can be easily distinguished by the leaf blade’s shape and indumentum, the number of the lateral veins, the shape of the corolla’s tube and lobes, and the length of stamens and staminodes (Table 1, Figs 1, 2, 3).

### Phylogenetic analysis

The aligned combined data matrix used in this analysis includes 1518 characters (ITS: 625; *trn*L-F: 893). They contained 490 parsimony informative and 796 variable sites. The best substitution models for *trn*L-F and ITS partitions, obtained via PhyloSuite v1.2.3, are GTR + G and SYM + I + G. Both ML and BI trees show the new species is most closely related to *Oreocharis acaulis* (Merr.) Mich.Möller & A.Weber, and *Oreocharis dinghushanensis* (W.T.Wang) Mich.Möller & A.Weber. (PP = 1.00, BS = 85%) with identical topologies (Fig. 2).

Phylogenetic analyses revealed congruent topologies between the maximum likelihood (ML) and Bayesian inference (BI) trees (Suppl. material 1: figs S1, S2). Therefore, the branch support values from both reconstructions—namely, posterior probabilities (PP) and bootstrap values (BS) were combined and presented on a Bayesian consensus tree (Fig. 2). The 62 samples of *Oreocharis* form a monophyletic clade. The two specimens of the proposed new species cluster together and are recovered as sister to *O. acaulis* and *O. dinghushanensis*, supporting the recognition of *O. zijinensis* as a new species.

### Taxonomic treatment

#### Oreocharis zijinensis

Taxon classificationPlantaeColeopteraCurculionidae

F.Wen, C.Xiong, K.Tan & Y.C.Xu sp. nov.

CDB1C689-A6BA-5605-8E30-1FB73E91846B

urn:lsid:ipni.org:names:77484425-1

Figs 3, 4

##### Diagnosis.

*Oreocharis zijinensis* resembles *O. acaulis* (Fig. 1A2, D2) in floral characteristics, including the shape and color of the corolla, and the number of the staminode. It is easily distinguished by its leaf blade elliptic to ovate apex acute, base attenuate to cuneate, slightly asymmetric, adaxially densely silvery-white pubescent, abaxially white villous especially on the veins, lateral veins 5–10, corolla tube cylindric to narrowly campanulate, slightly enlarged at the mouth, lobes equal, distinctly 2-lipped, apex acute, triangular semiorbicular, staminodes nearly equal.

**Figure 4.**
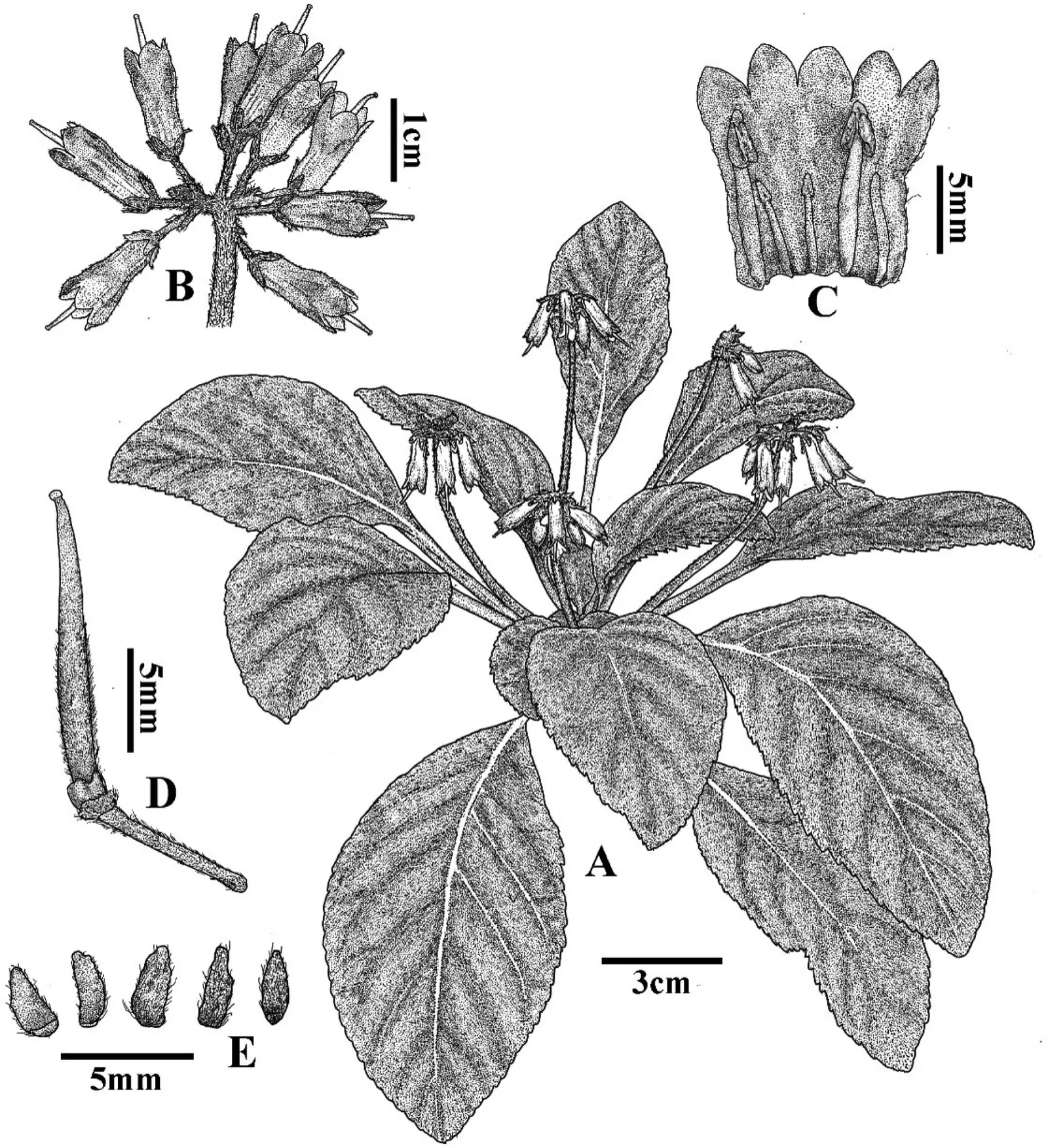
*Oreocharis zijinensis* sp. nov. **A**. Habit; **B**. Inflorescence; **C**. Opened corolla showing stamens; **D**. Pistil with disc; **E**. Calyx lobes. Drawn by Xiao-Mao Qian.

##### Type.

China. Guangdong: Heyuan City, Zijin County, Guzhu Town, Dongjiang Forest Farm, 23°36'N, 114°42'E, elev. ca. 118 m, April 25, 2024, *Ye-Chun Xu XYC210425-05* (holotype: IBK [IBK00473482!]; isotypes: IBK [IBK00473480! IBK00473481!]).

##### Description.

Perennial herbs, rhizomatous. Leaves in basal rosette, petiolate; petioles up to 5 cm long, 2–3 mm in diameter, white pubescent; leaf blade elliptic, oblong-ovate to ovate, 3–12 × 2–4 cm, adaxially green, densely silvery-white pubescent, abaxially pale green, white villous especially on the veins, apex acute, base attenuate to cuneate, slightly asymmetric, margin coarsely dentate-serrate; lateral veins 5–10 on each side of midrib, adaxially slightly sunken, abaxially protuberant. Inflorescence dichasial cyme, axillary, 1–9 per plant, each 1–10-flowered; peduncle 6–15 cm long, ca. 2 mm in diameter, white pubescent; bracts 2, light green, lanceolate, ca. 3 × 0.5–1 mm, white villous abaxially, glabrous adaxially, margin entire, apex acuate; pedicel light green, 0.5–1.5 cm long, ca. 0.5 mm in diameter, white villous. Calyx 5-parted to base, lobes equal, lanceolate to narrowly triangular, 3–4.5 × 1–2 mm, white villous abaxially, glabrous adaxially. Corolla pink at the apex and pale purple to white at the base, overall pale purple, 12–15 mm long, pubescent externally, glabrous internally, tube cylindric to narrowly campanulate, slightly enlarged at the mouth, 10–12 mm long, 3–5 mm in diameter; limb distinctly 2-lipped; lip 5-lobed, lobes equal, triangular semiorbicular, 2.5–3 mm long, 1.5–2 mm wide at the base, apex acute. Stamens 2, ca. 10 mm long, adnate to corolla from the base; filaments white, glabrous, straight, 7–8 mm long, ca. 1 mm in diameter, anthers brown, coherent in pairs, ca. 2.5 × 1.2–2.0 mm, divergent at base, confluent at apex; staminodes 3, nearly equal, linear, ca. 6.0 mm long, adnate to corolla from base. Disc waxy yellow, ca. 1.0 mm high, glabrous, margin undulate. Pistil glabrous at the apex and pubescent at the base, 1.4–1.6 cm long; ovary pubescent, cylindrical, 1–1.2 cm long, 1.0–1.5 mm in diameter; style glabrous, 3.0–5.0 mm long, 0.5–0.8 mm in diameter; stigma punctate, ca. 0.6 mm in diameter. Capsule linear, pubescent, 2–3 cm long.

##### Distribution and ecology.

The new species is endemic to Zijin County, central Guangdong Province. It grows on moist rock walls on both sides of streams under subtropical evergreen broad-leaved forests at an altitude of approximately 118 m in its type locality.

##### Phenology.

Flowering from March to April; fruiting from May to June.

##### Etymology.

The specific epithet ‘*zijinensis*’ refers to the type locality where the new species was found, Zijin County, Guangdong Province. The Chinese name is “Zǐ Jīn Mǎ Líng Jù Tái (紫金马铃苣苔)”.

##### Conservation assessment.

This new species is only found in the type locality and is presently known to occur in one population with ca. 800 matures individuals which were restricted to an area of 0.4 km^2^. The distribution area is currently within a protected area and is protected by local policies. Affected by drought in 2022, the population has shrunk slightly. Thus, following the IUCN (The International Union for Conservation of Nature and Natural Resources) guidelines (IUCN 2024), it is temporarily assessed as Vulnerable [VU].

##### Taxonomic affinities.

*Oreocharis zijinensis* morphologically resembles *O. acaulis* in having leaf blade ovate, margin denticulate, corolla pink, two fertile stamens, and punctate stigma. It can be easily differentiated from *O. acaulis* by several characteristics, including the shape and indumentum of the leaf blade, the number of the lateral veins, the shape of the corolla’s tube and lobes, and the length of stamens and staminodes. The detailed characteristics of the related species are provided in Table 1. The Bayesian 50% majority-rule consensus tree of *Oreocharis* inferred from the combined *trn*L-F and ITS sequence data reveals that *O. zijinensis* is phylogenetically most closely related to *Oreocharis acaulis* (Merr.) Mich.Möller & A.Weber (syn.: *Opithandra acaulis* (Merr.) B.L.Burtt) from Guangdong, China and *Oreocharis dinghushanensis* (W.T.Wang) Mich.Möller & A.Weber (syn.: *Opithandra dinghushanensis* W.T.Wang).

The morphological differences between *O. acaulis* and *O. zijinensis* are shown in the above text and Table 1. Morphologically, *O. dinghushanensis* can be readily distinguished from the new species by its leaf blades margin entire, indistinctly repand, leaf base broadly cuneate, and the corolla ca. 2.8 cm long; tube narrowly funnelform, ca. 2 cm long; staminodes 2, ca. 1.5 mm long; ovary linear; and stigmas 2, suboblong, glabrous.

## Supplementary Material

XML Treatment for Oreocharis zijinensis

## Additional information

### Artificial Intelligence (AI) use

The authors accept full responsibility for the content of the manuscript, including the disclosure of any use of AI.

No AI tools were used in the preparation of this manuscript.

### Author ORCIDs

G.-L. Zhu https://orcid.org/0009-0005-9556-2835

C. Xiong https://orcid.org/0000-0002-9082-2539

Y.-C. Xu https://orcid.org/0000-0002-2832-1514

J.-J. Tan https://orcid.org/0009-0007-3504-9727

Z.-B. Xin https://orcid.org/0000-0002-0062-6930

X.-M. Qian https://orcid.org/0009-0004-2335-022X

K. Tan https://orcid.org/0000-0002-9036-163X

F. Wen https://orcid.org/0000-0002-3889-8835

X.-X. Bai https://orcid.org/0000-0003-2449-6664

### Data availability

All of the data that support the findings of this study are available in the main text or Supplementary Information.

## Supplementary materials

Supplementary material 1 Data and results used in phylogenetic tree inference Data type docx https://binary.pensoft.net/file/1828084This dataset is made available under the Open Database License (http://opendatacommons.org/licenses/odbl/1.0/). The Open Database License (ODbL) is a license agreement intended to allow users to freely share, modify, and use this Dataset while maintaining this same freedom for others, provided that the original source and author(s) are credited. Guo-Li Zhu, Chi Xiong, Ye-Chun Xu, Jian-Jun Tan, Zi-Bing Xin, Xiao-Mao Qian, Ke Tan, Fang Wen, Xin-Xiang Bai
