# Supplementary material for: Oreocharis zijinensis (Gesneriaceae), a new species from Guangdong, China
Source: PhytoKeys. 2026 Nov 4;281:77–88. doi: 10.3897/phytokeys.281.204573 (PMC13755814; doi:10.3897/phytokeys.281.204573)
Supplement: Supplementary file 1 [file phytokeys-281-077_article-204573__-s001.docx]

**Data and results used in phylogenetic tree inference**

**Table S1:** The accession numbers of the sequences used in this study.

| *Species Name* | ITS | *trnL-F* | *Species Name* | ITS | *trnL-F* |
| --- | --- | --- | --- | --- | --- |
| *O. auricula* | DQ912664 | FJ501481 | *O. pankaiyuae* | HQ633021 | HQ632925 |
| *O. xiangguiensis* | HQ633027 | HQ632932 | *O. primuliflora* | HQ633019 | HQ632923 |
| *O. crispata* | MN164507 | MN164493 | *O. farreri* | JF697573 | JF697585 |
| *O. leiophylla (Bournea leiophylla)* | GU350644 | GU350676 | *O. dentata* | KM063156 | KM062916 |
| *O. nemoralis* | MN164498 | MN164490 | *O. henryana* | JF697574 | KM062918 |
| *O. lungshengensis* | HQ633013 | HQ632917 | *O. jinpingensis* | KM063163 | KM062923 |
| *O. magnidens* | HQ633026 | HQ632930 | *O. longifolia* | KM063152 | KM062912 |
| *O. burttii* | JF697570 | JF697582 | *O. rotundifolia* | KM063151 | KM062911 |
| *O. dalzielii* | JF697571 | JF697583 | *O. benthamii* | JF697572 | JF697584 |
| *O. curvituba* | MN164500 | MN164491 | *O. aurea* | HQ633016 | KM062914 |
| *O. esquirolii* | HQ633011 | HQ632915 | *O. nanchuanica* | KM063164 | KM062924 |
| *O. jiangxiensis* | HQ633029 | HQ632933 | *O. dinghushanensis* | GU350643 | GU350675 |
| *O. argyreia* | GU350639 | GU350671 | *O. acaulis* | HQ633012 | HQ632916 |
| *O. flavida* | MF315099 | MF315105 | *O. dasyantha* | MK587955 | MF315104 |
| *O. concava* | FJ501336 | FJ501505 | *O. hekouensis* | KM063174 | KM062934 |
| *O. convexa* | FJ501337 | FJ501506 | *O. mileensis* | KM062943 | KM063081 |
| *O. saxatilis* | KM063171 | KM062932 | *O. primuloides* | FJ501364 | FJ501546 |
| *O. speciosa* | KJ475420 | KM232646 | *O. pilosopetiolata* | MH629755 | MH629762 |
| *O. humilis* | GU350633 | GU350665 | *O. amabilis* | KJ475433 | KM232654 |
| *O. rhombifolia* | GU350632 | GU350664 | *O. chienii* | KM063148 | KM062908 |
| *O. rosthornii* | KM063167 | KM062927 | *O. cotinifolia* | HQ633010 | HQ632914 |
| *O.wangwentsaii(Ancylostemon mairei)* | GU350658 | GU350689 | *O. sinohenryi* | HQ633009 | HQ632913 |
| *O. delavayi* | KM063155 | KM062915 | *O. xieyongii* | MN783374 | MN786528 |
| *O. eximia* | KM063159 | KM062919 | *O. duyunensis* | MG722856 | MG722858 |
| *O. urceolata* | HQ633018 | HQ632922 | *O. stewardii* | HQ633022 | HQ632926 |
| *O. cinnamomea* | KM063161 | KM062921 | *O. ronganensis* | HQ633023 | HQ632927 |
| *O. cordatula* | KM063162 | KM062922 | *O. sinensis (Bournea sinensis)* | HQ633008 | HQ632912 |
| *O. craibii* | HQ633017 | HQ632921 | *M. peltata* | HQ632968 | HQ632872 |
| *O. begoniifolia* | HQ633025 | KM062926 | *A. biflora* | FJ501361 | FJ501541 |
| *O. dimorphosepala* | KM063165 | KM062925 | *A. bilirana* | MF446046 | HQ632891 |
| *O. georgei* | KM063157 | KM062917 | *A. paucipilosa* | HQ632990 | HQ632893 |
| *O. muscicola* | DQ912665 | FJ501548 | *A. clarkei* | FJ501360 | FJ501540 |
| *O. lancifolia* | HQ633020 | HQ632924 | *O.zijinensis 1* | PZ561624 | PZ884318 |
| *O.zijinensis 2* | PZ561625 | PZ884319 |  |  |  |

**Figure S1:** Maximum likelihood (ML) tree. The new species is shown in bold.

**Figure S2:** Bayesian inference (BI) trees. The new species is shown in bold.
